# Eliciting and Receiving Online Support: Using Computer-Aided Content Analysis to Examine the Dynamics of Online Social Support

**DOI:** 10.2196/jmir.3558

**Published:** 2015-04-20

**Authors:** Yi-Chia Wang, Robert E Kraut, John M Levine

**Affiliations:** ^1^Language Technologies InstituteSchool of Computer ScienceCarnegie Mellon UniversityPittsburgh, PAUnited States; ^2^Human-Computer Interaction InstituteSchool of Computer ScienceCarnegie Mellon UniversityPittsburgh, PAUnited States; ^3^Department of PsychologyUniversity of PittsburghPittsburgh, PAUnited States

**Keywords:** social support, health communication, self-disclosure, social media, support groups, emotions, natural language processing

## Abstract

**Background:**

Although many people with serious diseases participate in online support communities, little research has investigated how participants elicit and provide social support on these sites.

**Objective:**

The first goal was to propose and test a model of the dynamic process through which participants in online support communities elicit and provide emotional and informational support. The second was to demonstrate the value of computer coding of conversational data using machine learning techniques (1) by replicating results derived from human-coded data about how people elicit support and (2) by answering questions that are intractable with small samples of human-coded data, namely how exposure to different types of social support predicts continued participation in online support communities. The third was to provide a detailed description of these machine learning techniques to enable other researchers to perform large-scale data analysis in these communities.

**Methods:**

Communication among approximately 90,000 registered users of an online cancer support community was analyzed. The corpus comprised 1,562,459 messages organized into 68,158 discussion threads. Amazon Mechanical Turk workers coded (1) 1000 thread-starting messages on 5 attributes (positive and negative emotional self-disclosure, positive and negative informational self-disclosure, questions) and (2) 1000 replies on emotional and informational support. Their judgments were used to train machine learning models that automatically estimated the amount of these 7 attributes in the messages. Across attributes, the average Pearson correlation between human-based judgments and computer-based judgments was .65.

**Results:**

Part 1 used human-coded data to investigate relationships between (1) 4 kinds of self-disclosure and question asking in thread-starting posts and (2) the amount of emotional and informational support in the first reply. Self-disclosure about negative emotions (beta=.24, *P*<.001), negative events (beta=.25, *P*<.001), and positive events (beta=.10, *P*=.02) increased emotional support. However, asking questions depressed emotional support (beta=–.21, *P*<.001). In contrast, asking questions increased informational support (beta=.38, *P*<.001), whereas positive informational self-disclosure depressed it (beta=–.09, *P*=.003). Self-disclosure led to the perception of emotional needs, which elicited emotional support, whereas asking questions led to the perception of informational needs, which elicited informational support. Part 2 used machine-coded data to replicate these results. Part 3 analyzed the machine-coded data and showed that exposure to more emotional support predicted staying in the group longer 33% (hazard ratio=0.67, *P*<.001), whereas exposure to more informational support predicted leaving the group sooner (hazard ratio=1.05, *P*<.001).

**Conclusions:**

Self-disclosure is effective in eliciting emotional support, whereas question asking is effective in eliciting informational support. Moreover, perceptions that people desire particular kinds of support influence the support they receive. Finally, the type of support people receive affects the likelihood of their staying in or leaving the group. These results demonstrate the utility of machine learning methods for investigating the dynamics of social support exchange in online support communities.

##  Introduction

### Background

A high percentage of people with chronic or life-threatening diseases use online resources to obtain information about their condition and ways to cope with it. Although informational websites are the most popular, many people—especially cancer patients and survivors—participate in online health support communities [1,2]. A recent meta-analysis suggested that online support communities are effective in decreasing depression and increasing self-efficacy and quality of life [3]. Although several clinical trials suggest that participation in Internet-based support communities improves emotional well-being (eg, [4,5]), conclusions are ambiguous because most interventions have multiple components of which support group participation is only a part [6]. Moreover, research also shows that support interventions often do not provide the benefits they were designed to produce (eg, [7]). Thus, much remains to be learned about when and why support is effective in online communities.

Of the several categories of social support that have been identified in offline and online communities, emotional and informational support have received the most theoretical and empirical attention. Ridings and Gefen [8] reported that 76% of people who join online health communities do so to exchange emotional and informational support. Emotional support refers to the provision of caring, sympathy, or encouragement, whereas informational support refers to the provision of information or advice. Evidence in offline settings indicates that cancer patients seek out emotional support [9] and claim that it is especially helpful [10]. And research suggests that peer discussion focusing on emotional support enhances cancer patients’ psychological adjustment [11,12]. Participants in cancer support communities also exchange information and advice about diagnoses, treatments, adverse effects, relations with physicians, financial problems, and so on. And research suggests that information exchanged in offline support groups is associated with improvement in psychological well-being [13].

Interestingly, most research on social support, both offline and online, has focused on its physical and psychological effects, rather than on how it is elicited. For this reason, very little is known about the strategies that people use to seek support from others. This is surprising because the exchange of support is a dynamic communication process involving actions by support seekers and support providers [14]. During support elicitation, support seekers use a variety of techniques to indicate their need for support. During support provision, support providers recognize seekers’ behavior as requests for support and decide how to respond.

### Strategies for Obtaining Social Support

The Social Support Activation Model [15] categorizes support-seeking behaviors along 2 dimensions: (1) direct versus indirect elicitation and (2) verbal versus nonverbal elicitation. In online environments, where communication is verbal, a direct elicitation strategy might involve asking a question about a medication, whereas an indirect strategy might involve expressing fear about a diagnosis. Moreover, there is suggestive evidence that these 2 kinds of strategies may be used to elicit different kinds of support. For example, prior research suggests that participants in online support communities ask questions to get factual information [16] and that focused questions are more effective in getting useful answers than are open-ended ones [17]. In contrast, to elicit emotional support, participants often tell narratives about their disease and their emotional reactions to it [16,18]. The goal of this research is to further clarify the process by which members of online cancer support communities obtain informational and emotional support from other members of their group.

An important feature of our research is its reliance on automated techniques for analyzing a large number of conversational exchanges between community members. Most studies on communication in support communities are based on hand coding relatively small samples of messages (eg, [16,18-22]). Even Meier and colleagues’ [21] relatively ambitious effort only coded emotional and informational support in approximately 3000 messages. Because online support communities usually generate large numbers of posts, it is impractical to hand code all messages. Previous research has shown that it is feasible to partially automate some text analyses of conversations in online support communities (eg, [23,24]), but there has been little effort to fully automate the analysis of large numbers of messages in these communities.

Utilizing large-scale data and computer-aided content analysis is valuable for 3 reasons. First, because machine learning models can usually be easily replicated, they enable researchers interested in similar research questions to deploy comparable methods to challenge, verify, or extend others’ results. Second, the large-scale data that can be analyzed by machine learning methods allow researchers to answer more subtle research questions, conduct finer-grained analyses, and examine longer-term interaction patterns among participants. For example, using data from almost 60,000 exchanges from more than 30,000 participants in a breast cancer support community over a 9-year period, we have shown that being exposed to emotional and informational support has different effects on members’ subsequent participation in the group [25]. Third, computer-aided content analysis opens up opportunities for real-time interventions. For example, an online support community can use an automated model to detect the type of support an author of a particular post is seeking and then direct that person to discussions or other participants most relevant to his or her need.

In this paper, we use archival data to examine the relationships between the conversational moves people make in thread-starting posts and the amount and type of support they receive in response to them. In part 1, we conducted the analysis using a relatively small sample of approximately 1000 hand-coded conversational threads. In part 2, we replicated these results using machine learning algorithms to analyze more than 67,000 conversational threads. These algorithms use statistical procedures analogous to multiple regression to correlate language characteristics of messages with human judgments of them [26]. Specifically, part 2 describes techniques to automatically identify the extent to which messages exchanged in breast cancer discussion forums sought and provided emotional and informational support. Parts 1 and 2 test hypotheses suggested by, but not rigorously tested in, prior studies: (1) self-disclosure of support seekers elicits emotional support, whereas (2) asking questions elicits informational support. Finally, part 3 demonstrates additional benefits of automated coding by briefly reviewing previously published analyses showing that the type of support participants are exposed to predicts their continued participation in the group.

### Conceptual Model

**Figure 1:**
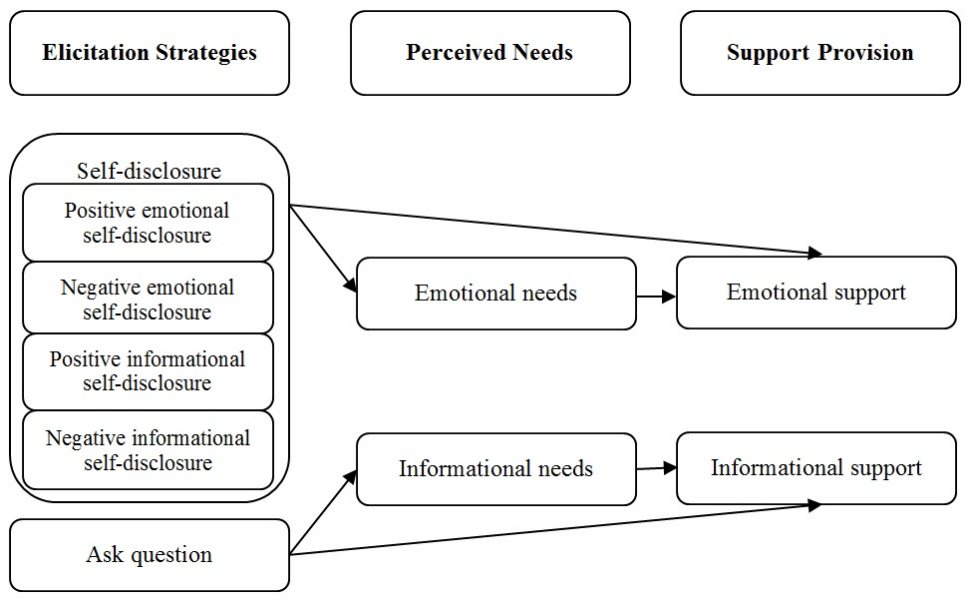
Conceptual model of social support elicitation and provision.

Figure 1 shows the model underlying our research. The model posits that different elicitation strategies on the part of support seekers (personal self-disclosure vs asking questions) produce different kinds of support from providers (emotional support vs informational support, respectively). Moreover, the model posits that this effect is mediated by the perceived needs of the support seeker. More specifically, the model posits that self-disclosure, irrespective of its content (emotional, informational) or valence (positive, negative), leads to perceived emotional needs, which in turn elicit emotional support. In contrast, asking a question leads to perceived informational needs, which in turn elicit informational support.

In this model, we hypothesize that the effects of elicitation strategies on support are mediated by the perceived needs of the support seeker. This hypothesis is important because, without such a process, a relationship between a seeker’s behavior and others’ responses could be interpreted as behavioral mimicry. Behavioral mimicry is a well-established phenomenon in human communication [27]; it occurs for both language (eg, [28]) and nonverbal behavior (eg, [29]), and it leads to better language understanding and interpersonal rapport. In conversations in support groups, behavioral mimicry could be confused with attempts to meet perceived needs. For example, most types of self-disclosure use first-person singular pronouns in constructions such as, “Well it’s been 2 weeks since my doctor called and gave me the results and I have been up and down since” or “I’m a 35 y/o happily married mother of 2 and completely and utterly shaken to my core.” People who give emotional support by replying with their own cancer experiences (eg, “I remember being in your place. I am only 4 months out from bilateral mastectomy and things have improved tremendously. Life will get better.”) are also likely to use first-person pronouns.

## Part 1: Using Human-Coded Data to Model How People Elicit Emotional and Informational Support

### Overview

Part 1 investigated the language strategies that support group members use to elicit emotional and informational support and the meditational role of their perceived needs. Because each of the constructs in Figure 1 is based on human judgments, this part of the study was limited to an analysis of approximately 1000 exchanges.

### Methods

#### Research Site

We studied support conversations in a large online breast cancer support community with a variety of communication platforms, including discussion boards and chat rooms. The discussion board platform was one of the most popular and active online breast cancer support communities on the Internet. At the time of our research, it had more than 90,000 registered members and 66 forums organized by criteria such as disease stage (eg, metastatic breast cancer), treatment (eg, hormonal therapy), demographic characteristics (eg, women aged 40-60 years), and treatment options (eg, breast reconstruction). In the forums, members ask questions, share stories, and read posts about how to deal with their disease. This discussion board platform is a rich environment for studying the dynamics of online support communities.

We collected all public posts on the discussion boards of the online support community from October 2001 to January 2011. During this period, the boards contained a total of 1,562,459 messages in 68,158 discussion threads. The median length of a discussion thread was 6 messages (mean 22.9, SD 280.5). The median life span of a thread, from the first thread-starting message to the last, was 2 days (mean 29.3, SD 115.6). A total of 81.12% (55,291/68,158) of thread-starting messages received a response within 24 hours, whereas 11.42% (7785/68,158) never received a response.

#### Measuring Elicitation Strategies, Perceived Support Needs, and Support Provision

We employed Amazon Mechanical Turk (MTurk) workers to judge how many messages from a random sample of 1000 thread starters and their first replies contained each of the constructs in Figure 1, such as negative informational self-disclosure or emotional support. MTurk is an online marketplace for crowdsourcing. It allows requesters to post jobs, called Human Intelligence Tasks (HITs), which workers, known as Turkers, can choose to perform. Snow et al [30] have shown that the combined judgments of a small number (between 5 to 7) of naïve judges on MTurk lead to ratings of texts that are very similar to those that experts make on such dimensions as emotions expressed, relative timing of events referred to, word similarity, word sense disambiguation, and linguistic entailment or implication.

We created independent tasks for judging each concept in Figure 1. One group of Turkers rated how many thread starter messages contained self-disclosure about events in the poster’s life or her thoughts and feelings. A second group rated how many thread starters asked questions. A third group rated how many thread starters sought emotional or informational support. Finally, a fourth group was shown the first replies to the thread starters (with the corresponding thread starters for reference) and judged how many provided informational and emotional support. The wording for the 9 judgments Turkers made are listed in Table 1. The Turkers made their judgments using a 7-point Likert scale (1=none; 7=a great deal).

**Table 1:** The tasks Turkers performed and the resulting intraclass correlation (ICC) for each construct.

Construct		ICC	Definition for Turkers	Prompt for Turkers
**Emotional self-disclosure**			Emotional self-disclosure is concerned with the extent to which the writer has discussed her feelings and emotions with others, such as happiness, fears, sadness, and anger.	
	Positive emotional self-disclosure	0.90	Example of positive emotional self-disclosure: “Now that chemo is done, I find myself waking up in the morning feeling a huge burden has been lifted from my shoulders.”	To what extent does this message contain positive emotional self-disclosure?
	Negative emotional self-disclosure	0.94	Example of negative emotional self-disclosure: “I am freaked out after reading my mammogram report.”	To what extent does this message contain negative emotional self-disclosure?
**Informational self-disclosure**			Informational self-disclosure is concerned with the extent to which the writer has discussed her personal information with others, such as health conditions, diagnosis results, and family status. Informational self-disclosure can be related to the positive, negative, or neutral life events of the writer.	
	Positive informational self-disclosure	0.85	Example of positive informational self-disclosure: “Took family to Cleveland Zoo for the first time in years and years.”	To what extent does this message contain positive informational self-disclosure?
	Negative informational self-disclosure	0.91	Example of negative informational self-disclosure: “I found a lump in my armpit about 5 weeks ago. It’s not fixed, but moveable. I have periodic tingling or single sharp pains in my left breast every once in a while.”	To what extent does this message contain negative informational self-disclosure?
**Asking a question**		0.91	When asking a question, the writer is requesting a response from the group. Questions can be asked directly and indirectly. Examples of questions: “What will you go through for a small chance to live longer?” “So I guess my question is, can a NOT dense breast, just average, have missed tumors on imaging?” “I am wondering if anyone has any advice on what I should do?” “Looking for any insight others may have on this.”	To what extent is this message asking a question?
**Eliciting support**				
	Emotional support elicitation	0.91	When seeking emotional support, the writer is trying to get understanding, encouragement, affirmation, sympathy, or caring.	To what extent is this message seeking emotional support?
	Informational support elicitation	0.95	When seeking informational support, the writer is trying to get advice, referrals, or knowledge.	To what extent is this message seeking informational support?
**Providing support**			There are 2 kinds of social support: emotional support and informational support.	
	Provide emotional support	0.92	Emotional support messages provide understanding, encouragement, affirmation, sympathy, or caring.	How much emotional support does this message provide?
	Provide informational support	0.92	Informational support messages provide advice, referrals, or knowledge.	How much informational support does this message provide?

Ten Turkers made each judgment, with different subsets rating each message. We aggregated workers’ responses for each message by averaging their ratings. Turkers were paid US $0.03 per message for judging question asking and US $0.05 per message for judging emotional self-disclosure, informational self-disclosure, perceived support needs, and support provision. To encourage workers to take the numeric-rating task seriously, they also highlighted words and phrases in the message that provided evidence for their ratings. To further control the annotation quality, we restricted the worker pool to Turkers who indicated a United States location and had at least of 98% their work accepted by their previous Turk employers.

Table 2 provides descriptive statistics and correlations among the 7 constructs judged by the Turkers.

**Table 2:** Descriptive statistics and correlations among constructs coded by Turkers.

Variable	Mean (SD)	1	2	3	4	5	6	7	8
1. Positive emotional disclosure	1.55 (0.96)	1							
2. Negative emotional disclosure	2.39 (1.52)	–.06	1						
3. Positive informational disclosure	1.89 (1.09)	.76	–.09	1					
4. Negative informational disclosure	3.58 (1.72)	–.17	.68	–.24	1				
5. Question asking	4.94 (2.17)	–.35	.06	–.34	.31	1			
6. Emotional support elicitation	2.75 (1.66)	.13	.79	.09	.58	–.11	1		
7. Informational support elicitation	4.21 (2.01)	–.36	–.06	–.37	.26	.88	–.28	1	
8. Provide emotional support	2.68 (1.43)	.16	.39	.14	.32	–.17	.49	–.24	1
9. Provide informational support	2.93 (1.47)	–.23	–.01	–.23	.17	.42	–.13	.49	–.17

Following are 2 examples from our final hand-coded dataset. Example 1 shows a message with high emotional support and low informational support and example 2 shows a message with low emotional support and high informational support. The example messages are lightly disguised using the techniques suggested by Bruckman [31]. Although the examples illustrate cases in which one type of support is high and the other low, across the full hand-coded sample, the two types of support were only weakly negatively correlated (*r*_972_=–.17, *P*<.001).

Example 1 (emotional support=5.7; informational support=1.0):

Julie-you have had such a difficult road, but yet you still manage to do well in school...I am truly inspired by you. Big cyber hugs and best wishes to you:>

Example 2 (emotional support=1.2; informational support=4.5):

Extranodal extension occurs when the tumor extends through the wall of the lymph node. This is noted on pathology reports, but in the main it isn’t very significant, and isn’t used in assessing cancer stage.

We assessed the reliability of raters’ judgments using intraclass correlations (ICC), which indicate the proportion of the variance in judgments that can be attributed to the message being judged. Table 1 shows the ICCs for the social support-related constructs used in this research. We validated Turkers’ judgments by comparing their judgments to expert judgments of the informational and emotional support contained in 50 messages selected from corpora studied by Meier et al [21] and Bambina [16]. The correlations between Turkers’ and experts’ average ratings for emotional support (*r*=.70) and informational support (*r*=.76) were both high.

### Results

#### Relationship Between Elicitation Strategies and Support Provision

**Figure 2:**
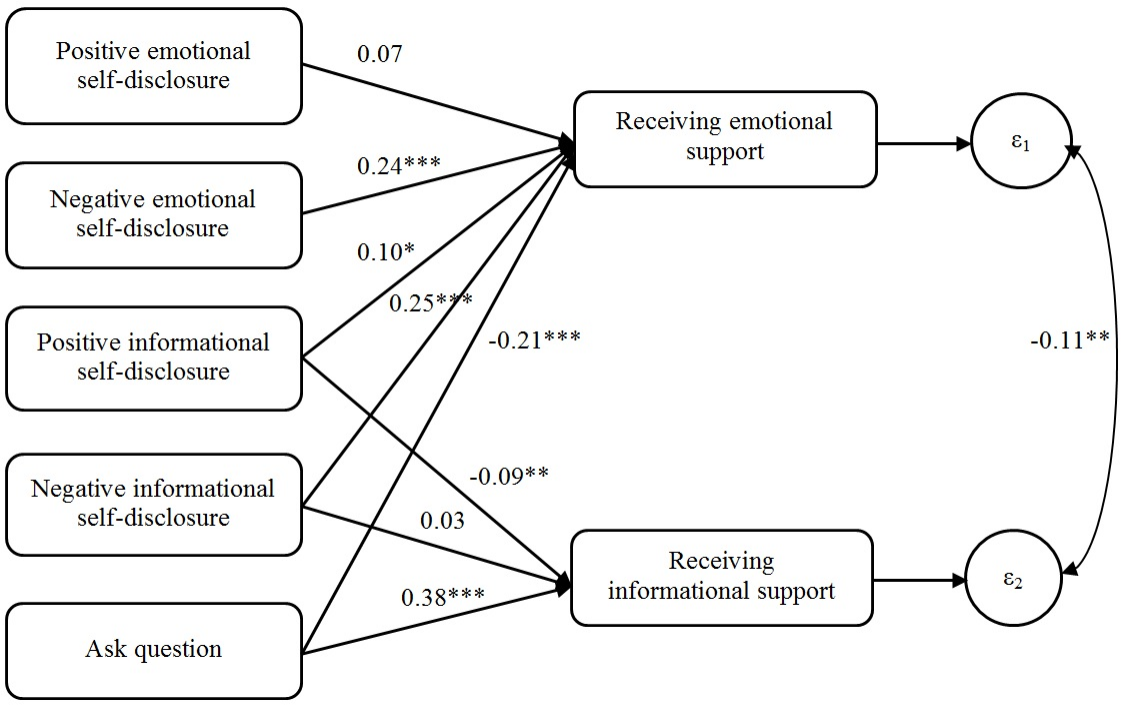
Path model showing the analysis of the social support communication process based on Turker-coded data. Values represent standardized regression coefficients. ε1 and ε2 represent error terms. * *P*<05; ** *P*<01; *** *P*<001.

We first tested the hypotheses that self-disclosure elicits emotional support whereas questions elicit informational support using the dataset of 1000 Turker-coded messages. We used structural equation modeling to examine the relationships between the amount of self-disclosure and question asking in the thread starters and the amount of emotional and informational support provided in the first reply. Standardized regression coefficients (β) are reported. Figure 2 is the path model showing the results. This model fits the human-coded data well, with the comparative fit index (CFI)=0.98 and the root mean squared error of approximation (RMSEA)=0.062 (see [32] for rules-of-thumb for evaluating the goodness of fit for structural equation models; a CFI>0.97 is generally considered a good fit, whereas a RMSEA between 0.05 and 0.08 is generally considered an acceptable fit). Note that we did not include the relationships between positive/negative emotional self-disclosure and informational support provision in the model because these relationships were not significant and excluding them improved the model fit. This applied to all the models reported in this paper.

The more thread-starting messages contained negative emotional self-disclosure (beta=.24, SE 0.04, *P*<.001) and negative informational self-disclosure (beta=.25, SE 0.04, *P*<.001), the more responses to them provided emotional support. Positive informational self-disclosure was also associated with more emotional support in replies, although not as strongly (beta=.10, SE 0.04, *P*=.02). However, positive emotional self-disclosure was not significantly associated with more emotional support (beta=.07, SE 0.04, *P*=.10). In contrast to these effects of self-disclosure which generally increased emotional support, asking questions was associated with receiving less emotional support (beta=–.21, SE 0.03, *P*<.001).

Participants used different strategies to elicit informational support. Thread starters were more likely to get information and advice when they explicitly asked for it, but less likely to receive it when they described positive events in their lives. The more the thread starters asked questions, the more the first reply provided informational support (beta=.38, SE 0.03, *P*<.001). In contrast, when the thread starter revealed more positive informational self-disclosure, the reply contained less informational support (beta=–.09, SE 0.03, *P*=.003). Negative informational self-disclosure was not associated with receiving informational support (beta=.03, SE .03, *P*=.306). These results are consistent with prior research indicating that asking explicit questions and providing a rationale elicited information and advice [33,34]. In contrast, when thread starters described positive events in their lives, they were less likely to receive information and advice, even if they asked questions. Perhaps in these cases, recipients believed that the thread starter did not really need their help, although asking for it.

#### Mediation Effect of Perceived Support Needs

When people started a thread with self-disclosure and especially when they revealed negative thoughts and feelings and negative events in their lives, and when they refrained from asking questions, others were likely to offer them emotional support. However, when they asked questions and refrained from describing positive events in their lives, others offered them information and advice instead. We assumed these language features lead to social support because other participants in the community perceived them as indicators of a need for emotional support and informational support, respectively. That is, other members of the community treated these language features as requests for particular kinds of support. To test this assumption, we conducted mediation analysis to assess whether the language features in thread-starting messages have their effects on the emotional and information support in replies because they signaled a desire for a particular kind of support.

**Figure 3:**
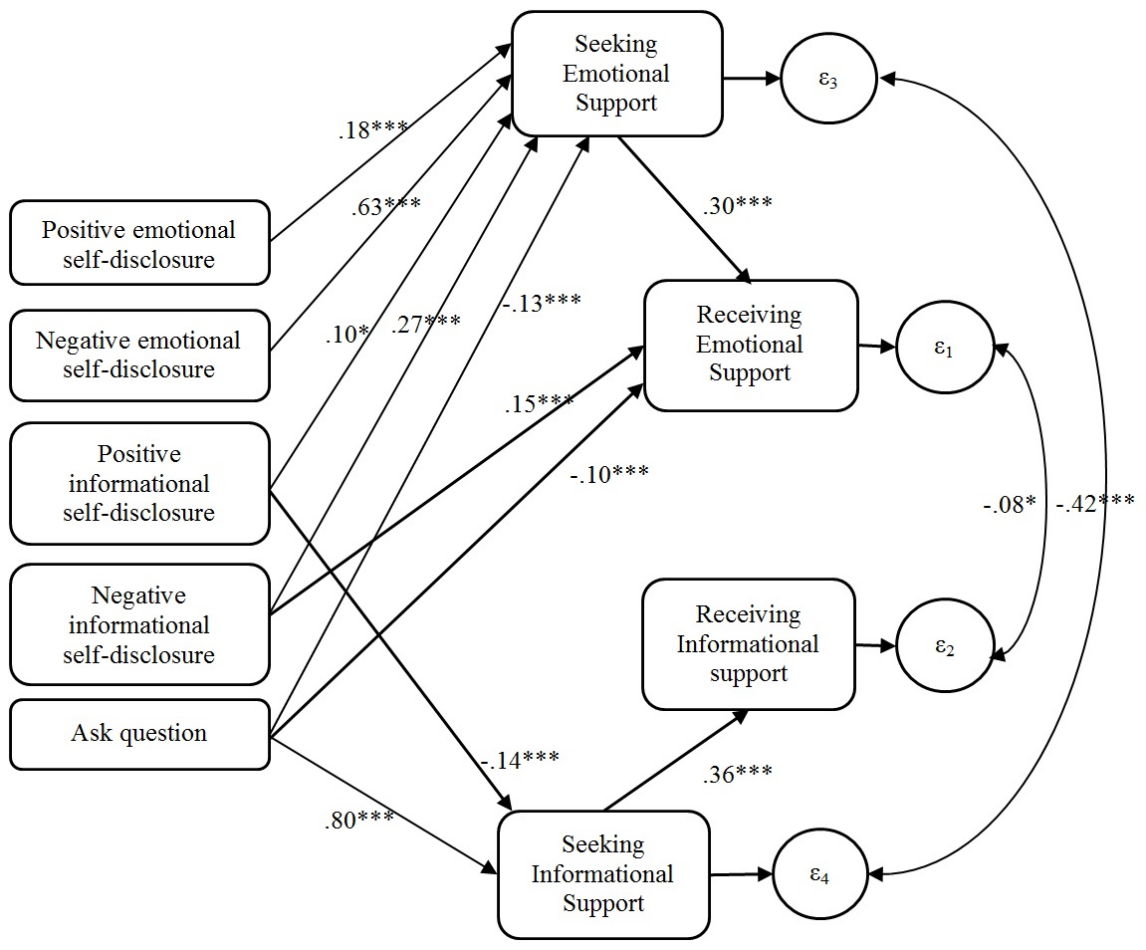
Direct effects of language features in a thread-starting post on the perception that the poster was seeking emotional and informational support and on the receipt of emotional and informational support. Values represent standardized regression coefficients. ε1, ε2, ε3, and ε4 indicate error terms. * *P*<.05; ** *P*<.01; *** *P*<.001.

Results are shown in Figure 3, which includes only the direct paths between constructs. Although the CFI index (0.98) indicated that the mediation model was a good fit to the data, the RMSEA criterion (0.119) was problematic. As Schermelleh et al [32] noted, “it is quite difficult to decide on data-model fit or misfit, especially if various measures of model fit point to conflicting conclusions about the extent to which the model actually matches the observed data...[Although rules-of-thumb exist about what constitutes a good fitting SEM model], these rule-of-thumb cutoff criteria are quite arbitrary and should not be taken too seriously.” We interpret our findings as indicating mediation. As Figure 3 illustrates, people received emotional support when they were perceived as seeking it (beta=.30, SE 0.04, *P*<.001) and informational support when they were perceived as seeking that (beta=.36, SE 0.04, *P*<.001). The effects of the language features that predicted receiving either informational or emotional support in Figure 2 were all at least partially mediated by the perception that the author was seeking this particular kind of support, as shown in Figure 3. The data came from human judgments of each construct. Numbers represent standardized regression coefficients from a structural equation model. Only direct links between constructs significant at *P*<.05 are shown. Indirect effects can be calculated by multiplying the direct effects (eg, the indirect effects of asking questions on receiving informational support mediated by seeking informational support is .80*.36=.29).

Focusing on the receipt of emotional support, the effects of all the speech acts were either partially or completely mediated by their influence on judgments that the writer was seeking emotional support. Negative emotional self-disclosure (writing about sadness, fear, anger, and other negative thoughts and feelings) was associated with receiving more emotional support, and this effect was completely mediated by the perception that the writer was seeking emotional support (indirect effect/total effect=.19/.21=90%); the direct effect of negative emotional self-disclosure on getting emotional support became insignificant after controlling for the perception that the writer was seeking emotional support (beta=.02, *P*=.74). Similarly, the effect of positive informational self-disclosure on receiving emotional support was partially mediated by judgments that the writer was seeking emotional support (.03/.13=23%), leaving a nonsignificant direct path (beta=.10, *P*=.07). The effect of negative informational self-disclosure on receiving emotional support was also partially mediated by judgments that the writer was seeking emotional support (.08/.23=35%). The direct effect of negative informational self-disclosure on receiving emotional support decreased from .25 (in Figure 2) to .15 (*P*<.001) when controlling for the perception that the writer was seeking emotional support. The negative effect of asking questions on receiving emotional support was also partially mediated by the judgment that the writer was not seeking emotional support (–.04/–.14=29%). However, asking questions also had a direct negative effect on receiving emotional support (beta=–.10, *P*<.001).

Focusing on the receipt of informational support, the effects of positive and negative informational self-disclosure and asking questions were completely mediated by their direct influence on judgments that the writer was seeking informational support. Although people were likely to receive informational support after asking questions (total effect=.26), the total effect was mediated by the link between asking questions and seeking information and between seeking information and receiving it (.29/.26=112%). Similarly, 42% of the total negative association of positive information disclosure on receiving informational support was mediated by its association with the perception the writer was seeking informational support (–.05/–.12=42%).

## Part 2: Using Machine-Coded Data to Model How People Elicit Emotional and Informational Support

### Methods

#### Overview

Part 2 sought to partially replicate the analyses previously described, which were based on human coding of 1000 interactions, by using machine learning techniques to automate the coding of 58,357 interactions. We constructed machine learning models to automatically measure both the language elicitation strategies posters used in their thread-starting posts and the emotional and informational support they received in the first reply to them. Our goal was to use machine learning approaches to correlate characteristics of messages with human judgments made about various elicitation strategies and the presence of emotional and informational support. As described subsequently, the input features included language characteristics of the messages, such as their length, presence of words from general and domain-specific dictionaries, syntax features (eg, the ordering of noun and verb phrases), higher-level semantic features (eg, the presence of advice and questions), and questions. The outputs were numerical values representing the extent to which a message asked questions or provided various types of self-disclosure or support. A model was successful if (1) its assessments of the outcomes of interest matched those produced by human judges and (2) it was parsimonious, using a small number of input features while producing an accurate assessment.

Building, validating, and applying the machine learning models involved 5 steps. First, human judges hand-coded language strategies and support provisions in a sample of messages. Their judgments represented the “ground truth” or “gold standard” to which we compared the machine learning estimates. Second, we represented the messages as a set of language features that could be interpreted by computers and would be the input to the machine learning algorithms. Third, we identified the best machine learning models from a part of the hand-coded data by iteratively testing different configurations of machine learning algorithms and combinations of input features. Fourth, to evaluate the performance of the final models, we applied them to a holdout sample of data. Finally, we tested hypotheses by applying the machine learning models to automatically code the 58,357 thread starter–first reply pairs in the entire dataset.

#### Building Machine Learning Models for Social Support Concepts

Because most health researchers will not be familiar with the methods used to construct the machine learning models to measure self-disclosure, question asking, and emotional and informational support, we will describe them and their rationale in some detail. For a general treatment of machine learning, we refer the reader to Witten et al [35]. The processes for building models for each construct in our model were similar, so we illustrate the general approach by describing how to measure the presence of emotional and informational support.

##### Step 1: Creating the Human-Coded Dataset

We used the annotations collected in part 1 as the human-coded dataset for machine learning model training. The details regarding how we obtained the human annotations were described in part 1.

##### Step 2: Identifying Language Features

In the second step, messages were represented as lower-level, computer-readable language features, which were likely to provide cues that a text was self-disclosing, asking a question, or offering emotional or informational support. We focused our search for language features in domains that we believed would reflect the topics and writing styles people use when they talk about their disease and their relationship to it. We focused on the 6 language domains:

1. Body: one of the most common conversational themes in online health support communities involves patients’ bodies and how disease changes their bodies. Participants might describe their bodily conditions to look for advice or to share their experiences with others. Sometimes they tell others the results of their treatment by mentioning changes in their bodies, such as weight loss or the size of a tumor. The discussion of the body can signal the offering of support and not just self-disclosure.
2. Positive/negative emotions and events: revealing underlying emotions or sharing personal life events and stories is a way to self-disclose and provide support. Online support participants often share personal feelings about themselves and use language that indicates happiness, fear, anger, and other emotional states. People also often describe positive things that happened in their lives, such as successful treatment, to encourage others and provide support to them.
3. Social: social relations are crucial for cancer survivors. Their disease may change how they interact with others. Through interaction with others, they can feel supported and gain the strength to face their disease. In the absence of support, they may feel abandoned.
4. Spiritual: spiritual or religious comments can provide social support, especially for those with strong religious beliefs. Examples of spiritual comments in this online breast cancer community are “God bless” and “I’ll add you to my prayer list.”
5. Time: time-related information is often mentioned in online support discussions. For instance, cancer stages and treatment processes involve references to time.
6. Language structures: language structures, such as sentence structure and punctuation, can be indicators of social support. For example, sentences that start with the pronoun “I” are likely to be self-disclosing. And when a writer is asking direct questions, the sentences often end with question marks or use a verb-subject order (eg, “can you”).

To operationalize the linguistic features in the 6 domains, we measured 3 kinds of low-level language features as summarized in Textbox 1.

**Summary of the 3 kinds of basic language features.**

1. Linguistic Inquiry and Word Count (LIWC) dictionaries

- Pronoun: I, we, you, she/he, they, impersonal pronoun
- Tense: auxiliary verb, past, present, future
- Emotion: positive emotion, negative emotion (anxiety, anger, sadness)
- Other topics: cognitive mechanism, biological processes, time, religion, death

2. Latent Dirichlet allocation (LDA) topical dictionaries

- Prediagnosis, treatment plan, forum communication, adjusting to diagnosis, financial concerns, lymphedema, diet, family and friends, positive life events, surgery, thoughts and feelings, chemoradiation, family history, emotional reaction, tumor treatment, spiritual, emotional support, routine and schedule, hair loss and appearance, postsurgery problems

3. Syntactic and sentiment features

- Length: sentence count, word count per sentence
- Negation: not
- Part-of-speech: proper nouns, adjectives, cardinal numbers
- Advice pattern: advice verbs, <Please + VERB>, <If + you>, <You + MODAL>
- Question pattern: question marks, any, <VERB + SUBJECT>, indirect questions
- State: be verbs, stative verbs, <SUBJECT_I>, <SUBJECT_I + positive_ADJECTIVE>, <SUBJECT_I + negative_ADJECTIVE>
- Subjectivity: strong subjectivity, weak subjectivity
- Drug: FDA drugs

The first type of language feature was derived from generic dictionaries developed by Pennebaker and colleagues [36] in the Linguistic Inquiry and Word Count (LIWC) program, which measures function words (especially various types of pronouns) and topics with psychological relevance (eg, positive emotion words, negative emotion words, cognition words). Second, we created specialized, cancer-related dictionaries using latent Dirichlet allocation (LDA) topic modeling. Third, we included syntactic features of the messages. Finally, because storytelling is itself a complicated language feature, we included a feature that indicates whether a message reveals a story about the writer.

The LIWC program is a word counting program that calculates the frequency with which words in a text match each of 68 predefined dictionaries representing linguistic dimensions (eg, pronouns, tense), psychological constructs (eg, positive emotion), and personal concerns (eg, leisure, death) [36]. Alpers and colleagues [23] analyzed several hundred posts in an online breast cancer community using a human rater and LIWC and demonstrated a moderate correlation between the ratings assigned by the rater and LIWC scores. Motivated by their work, we included LIWC scores in our machine learning models and considered them as baseline features. LIWC dictionaries were selected based on their a priori relevance to social support concepts. For example, for emotional support, words from the we dictionary (eg, “we,” “us,” “ours”) express feeling of companionship and solidarity, whereas those from the positive emotion (eg, “love”) and religion dictionaries (eg, “pray”) express encouragement. For informational support, words form the impersonal pronoun dictionary (eg, “it”) and the present tense dictionaries are often used to describe objective facts. We included 17 LIWC dictionaries listed in Textbox 1.

The preceding features are generic rather than tailored to the content of cancer-related discussions. Research on analyzing text in support communities suggests that different topics can signal different types of social support interactions [37]. For example, when people use surgery-related terms, such as reconstruction, skin, and surgeon, they are likely to be seeking information, but when people express their fears and other feelings, they are likely to be seeking emotional support. LDA is a statistical generative model that can be used to discover hidden topics in documents and the words associated with each topic [38]. We first trained an LDA model using 30,000 breast cancer messages randomly selected from the entire dataset. The model was set to derive 20 latent topics. For each topic, we chose the 500 words with the highest association with the topic and used them to build a topic dictionary. Two experts familiar with cancer manually assigned a label to each topic (see Table 3). Examples of topics derived from the LDA analysis include emotional reaction (eg, “better,” “lucky,” and “sacred”), diet (“fat,” “weight,” “food,” “exercise,” and “body”), and tumor treatment (eg, “biopsy,” “nodes,” “positive,” and “report”). Textbox 1 lists the 20 topics included in the LDA analysis and Table 3 also shows the words that are most representative of each LDA topic dictionary. Each LDA topical feature calculates the frequency of words in a message matching its corresponding dictionary. When the LDA procedure identified topics that overlapped with preexisting LIWC dictionaries (eg, the LDA family/friends dictionary overlaps the LIWC friends and family dictionaries), we used only one of the dictionaries in the machine learning models for reasons of parsimony.

**Table 3:** Samples of vocabulary in latent Dirichlet allocation (LDA) topic dictionaries.

LDA topic	Sample vocabulary
Prediagnosis	Told, appointment, wait, back
Treatment plan	Clinical, risk, medicine, therapy
Forum communication	Post, read, help, thread
Adjusting to diagnosis	Understand, trying, experience
Financial concerns	Insurance, plan, company, pay
Lymphedema	Arm, pain, swelling, fluid, area
Diet	Eat, weight, food, exercise, body
Family/friends	Daughter, sister, wife
Positive life events	Love, nice, happy, enjoy, fun
Surgery	Breast, surgeon, mastectomy
Thoughts/feelings	Think, remember, believe
Chemoradiation	Chemo, radiation, treatment
Family history	Mom, children, age, young
Emotional reaction	Better, lucky, scared
Tumor treatment	Biopsy, nodes, positive, report
Spiritual	Love, god, prayer, bless, peace
Emotional support	Hope, hug, glad, sorry, best, luck
Routine/schedule	Today, night, sleep, work
Hair loss/appearance	Hair, wig, grow, head
Postsurgery problems	Pain, blood, tamoxifen, symptom

Sentence count and word count per sentence were features designed to represent the length and complexity of messages. The negation feature is the number of sentences in a message containing negation words or phrases, such as “not,” “shouldn’t,” or “did not.” Because some parts of speech (POS) can signal disease-relevant information or emotion, we counted the number of several specific POS tags. For instance, professional labels can be signaled as proper nouns (eg, “Dr. Smith”), emotional states can be signaled by adjectives (eg, “happy” life), and numbers can be used to describe symptoms or treatments (eg, “10 days since the biopsy”). We applied the Stanford POS tagger [39] to assign POS tags for words and extracted relevant POS features. To identify sentences involving requests and advice, we identified several text patterns or verbs in messages. For instance, <Please+VERB> is a pattern that detects sentences beginning with the word “please” followed by a verb (eg, “Please give”). <You+MODAL> is a pattern that counts the number of sentences that start with a pronoun “you” and are immediately followed by a modal verb expressing possibilities (eg, “should,” “might,” “must”). Furthermore, the advice verbs feature considered the occurrence of verbs such as “make,” “suggest,” and “wish.” The question pattern features were designed to count the number of both direct and indirect question sentences in a message. There were 4 question pattern features, including count of question marks, count of indefinite pronouns (eg, “any,” “anyone,” “anybody”), sentences starting with a modal verb (eg, “Does anyone know...”), and indirect questions (eg, “I am wondering if...”). The state features were designed to capture states that last for a while. These features are potentially related to description of emotional states and storytelling. We counted the number of “be” verbs (eg, “My mom is depressed”), stative verbs (eg, “I love my mom”), sentences starting with the subject “I” (ie, <SUBJECT_I>), and sentences starting with the subject “I” followed by positive or negative adjectives (ie, <SUBJECT_I + positive_ADJECTIVE> and <SUBJECT_I + negative_ADJECTIVE>). Sentiment features described the subjectivity of a text segment. We counted the number of strong subjectivity words (eg, “reject,” “nervous”) and weak subjectivity words (eg, “idea,” “suggest”) for every message using the OpinionFinder subjectivity lexicon [40]. Finally, the number of drug terms in each message was counted, based on an exhaustive list of medicine names collected from the Food and Drug Administration website [41].

The binary story feature indicated whether or not the writer was telling a personal story in the message. A linguistic expert categorized 2200 randomly selected messages as a story or not a story. We then built a machine learning model to predict whether the writer of a message was or was not telling a story. The feature set for this model included all the basic language features described previously. The kappa agreement statistic between human coding and computer prediction was .88, which indicates high agreement [42]. We then applied the story model to detect story messages in the 1.5 million messages collected from the support community. In Table 4, the basic language features are mapped onto the language usage domains.

**Table 4:** Mapping of language features onto language usage domains.

Language features and usage domains	LIWC	LDA topics	Syntax
Body	Biology processes, death	Prediagnosis, treatment plan, adjusting to diagnosis, lymphedema, diet, surgery, chemo radiation, family history, tumor treatment, hairloss and appearance, postsurgery problems	Drug
Positive/negative emotions and events	Sentiment, cognitive mechanism	Financial concerns, positive life events, thoughts and feelings, emotional reaction, emotional support	State, subjectivity
Social	Pronouns	Forum communication, family and friends	—
Spiritual	Religion	Spiritual	—
Time	Time	Routine and schedule	—
Language structure	Tense	—	Length, negation, part-of-speech, advice pattern, question pattern

##### Steps 3 and 4: Construction and Performance of Machine Learning Models

Our task was a machine learning regression problem. We built 7 machine learning regression equations to predict in each message the mean of the Turkers’ judgments of the amount of emotional or informational support, question asking, or self-disclosure (see the judgment dimensions listed in Table 5). The predictor variables were the dictionaries and other features listed in Textbox 1 and the story feature. We used Weka, a machine learning toolkit, to build the support vector machine regression models (SMOreg) [35]. The 1000 thread starters or their first replies coded by MTurk workers were randomly partitioned into a training set (80%), a development set (10%), and a test set (10%). The training set was used to build the models. The development set was used to evaluate the accuracy of different configurations of the models and variations in the features used. Once the models achieved good performance on the development data, we used the test set to evaluate how well the final regression equations performed. We evaluated the predictions using the Pearson product moment correlation between the human-coded ratings and machine measurements for the 100 messages in the test sample. The agreement between the human-coded ratings and machine measurements was .65 averaged across the 7 dimensions and ranged from .85 for informational support provision to .44 for positive emotional self-disclosure. Table 5 shows the evaluation results for each support-related construct.

**Table 5:** Accuracy and features for 7 machine learning models.

Machine learning model and top 10 features		Accuracy (Pearson *r*)^a^	Feature weight of SMOreg^b^
**Positive emotional self-disclosure**		.44	
	Positive emotion		0.32
	Word count per sentence		0.28
	Religion		0.25
	<Please + VERB>		–0.21
	Sentence count		0.16
	<SUBJECT_I + positive_ADJECTIVE>		0.13
	Negation		–0.10
	We		0.07
	Financial concerns		–0.07
	Strong subjectivity		0.07
**Negative emotional self-disclosure**		.59	
	Anxiety		1.18
	Anger		0.51
	<SUBJECT_I>		0.40
	Sadness		0.28
	<SUBJECT_I + negative_ADJECTIVE>		0.27
	Death		0.23
	Negation		0.18
	Strong subjectivity		0.17
	Word count per sentence		0.14
	Sentence count		0.14
**Positive informational self-disclosure**		.45	
	Positive emotion		0.31
	Religion		0.27
	Sadness		–0.25
	Sentence count		0.25
	Word count per sentence		0.23
	<Please + VERB>		–0.20
	<SUBJECT_I + positive_ADJECTIVE>		0.16
	Routine and schedule		0.13
	Biological processes		–0.13
	Auxiliary verb		–0.12
**Negative informational self-disclosure**		.64	
	Anxiety		0.42
	Sentence count		0.41
	Any		0.32
	Biological processes		0.28
	Tumor treatment		0.26
	<SUBJECT_I>		0.26
	<SUBJECT_I + positive_ADJECTIVE>		–0.25
	Anger		0.24
	I		0.23
	Lymphedema		0.21
**Question asking**		.78	
	Sentence count		–0.82
	Religion		–0.72
	Word count per sentence		–0.64
	Positive emotion		–0.59
	Question marks		0.52
	Any		0.50
	Proper nouns		–0.40
	<Please + VERB>		0.36
	Spiritual		–0.30
	Negation		0.27
**Emotional support provision**		.81	
	Sentence count		0.55
	Emotional support		0.46
	We		0.45
	She/He		–0.44
	You		0.37
	Question marks		–0.33
	Strong subjectivity		0.24
	Adjusting to diagnosis		0.23
	Be verbs		0.23
	Positive life events		–0.23
**Informational support provision**		.85	
	Sentence count		1.13
	Word count per sentence		0.38
	Question marks		–0.33
	Spiritual		–0.26
	Postsurgery problems		0.22
	I		–0.20
	<If + you>		0.20
	Strong subjectivity		–0.19
	Forum communication		–0.17
	Tumor treatment		0.16

^a^ The accuracy correlation is the Pearson product moment correlation between the average of 10 human judgments and the output of the machine learning model.

^b^ The output feature weight of the support vector machine regression model shows the strength of the association between the presence of a feature in a message and human judgments of that message.

Table 5 also presents the 10 most important features associated with each model. Each number in the table represents an output weight of the support vector machine regression model showing the strength of the association between human judgments and machine predictions. For example, the occurrence of religion words from LIWC was a positive indicator of positive emotional self-disclosure (weight=.25) but a negative predictor of asking questions (weight=–.72). In Table 5, features listed in angle brackets indicate patterns (eg, SUBJECT_I indicates a pattern with the pronoun “I” in the subject position in the sentence), whereas other features are names of LIWC or LDA-derived dictionaries.

Given the adequate validity of all these models, we then applied them to measure the amount of emotional and informational support, self-disclosure, and question asking for each of the 1.5 million messages in our dataset (Step 5).

### Results

#### Machine-Coded Versus Human-Coded Data

Using machine-coded data from 58,357 thread starters that received at least 1 reply, we sought to partially replicate the analyses presented in part 1 (Figure 2) which tested the hypotheses that self-disclosure elicits emotional support whereas questions elicited informational support. We did not attempt to replicate the mediation analysis from Figure 3 using machine-coded data because the lower-level language features predicting perceived support needs would be the same as those predicting self-disclosure and question asking, producing problems of common method variance. Table 6 shows the descriptive statistics and correlations among the variables used in the model.

**Table 6:** Descriptive statistics and correlations among constructs using machine learning (N=58,357 discussion threads).

Variable^a^	Mean (SD)	1	2	3	4	5	6
1. Positive emotional disclosure	1.27 (0.26)	1					
2. Negative emotional disclosure	2.29 (1.08)	.19	1				
3. Positive informational disclosure	1.67 (0.41)	.83	.14	1			
4. Negative informational disclosure	3.69 (1.26)	–.05	.67	.04	1		
5. Question	5.26 (1.29)	–.53	.16	–.48	.50	1	
6. Provide emotional support	2.64 (1.06)	.24	.23	.24	.17	–.13	1
7. Provide informational support	2.95 (1.18)	–.22	.08	–.20	.21	.36	–.16

^a^ All variables were estimates from machine learning models predicting the amount a message contained the constructs in the first column, rated on a 7-point Likert scale where 1=not at all and 7=very much. The self-disclosure and question measures were based on the thread-starting message, whereas the measures of emotional and informational support were based on the first reply received in the thread.

**Figure 4:**
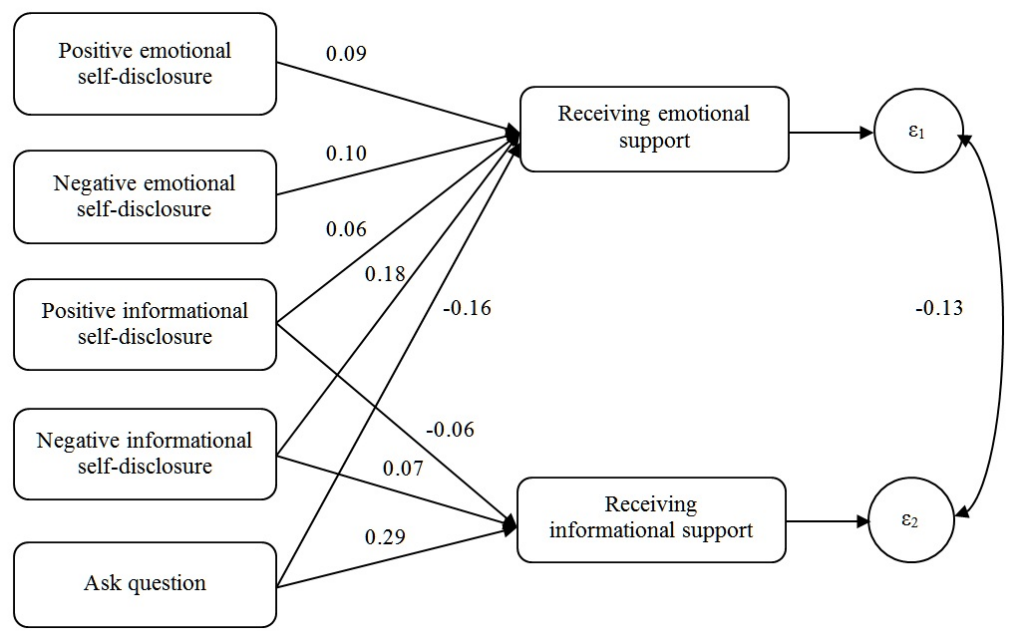
Path model showing the analysis of the social support communication process based on machine-coded data. ε1 and ε2 indicate error terms. All P values <.001.

Figure 4 is the path model showing the results in which the language features in the thread-starting posts were used to predict the amount of emotional and information support provided in the first reply. The results reported are similar to those obtained if we used the average amount of support provided in the first 5 replies. This model fit the data very well according to both the CFI (CFI=0.998) and the RMSEA (RMSEA=0.016).

Most results from this large sample of machine-coded data replicated those from the small sample of human-coded data. The model showed that the more any of the 4 types of self-disclosure occurred in the thread-starting message, the more the first reply contained emotional support (all *P*<.001), although the effect for positive emotional self-disclosure was not significant (*P*=.10) in the model of human-coded data. Specifically, in eliciting emotional support, the effect of negative informational self-disclosure (beta=.18, SE 0.01) was stronger than the effect of either positive emotional self-disclosure (beta=.09, SE 0.01) or negative emotional self-disclosure (beta=.10, SE 0.01), which in turn were stronger than the effect of positive informational self-disclosure (beta=.06, SE 0.01). However, these comparisons of effect strength should be treated with caution given high correlations among some constructs in Table 6 (eg, the correlation between positive emotional and positive informational self-disclosure was .83). As with the human-coded dataset, when the thread starter asked questions, the reply contained less emotional support (beta=–.17, SE 0.01) but more informational support (beta=.29, SE 0.01). Positive informational self-disclosure seemed to depress informational support (beta=–.06, SE<0.00). Negative informational self-disclosure seemed to elicit informational support (beta=.07, SE<0.00), although this association was not significant in the human-coded dataset, presumably because of the small sample size.

### Discussion

Results from both parts 1 and 2 are consistent with the thesis that different conversational strategies lead to receiving different types of social support. People received information and advice when they explicitly requested it, whereas they received emotional support when they disclosed more details about events in their lives and their thoughts and feelings, especially negative ones. Using distinct methods—a small sample of hand-coded data and a large sample of machine-coded data—these parts of the research reached similar conclusions about the relationship between language people used in soliciting support and the kind of support they received. However, conclusions drawn from machine learning models can be more precise because of their larger sample sizes. For example, the sign for the influence of positive emotional self-disclosure on receiving emotional support was the same in the human-coded and machine-coded datasets but only achieved statistical significance in the latter because of the greater statistical power associated with a much larger sample of conversations. These results suggest that machine coding of conversational data can add value beyond what can be learned from human-coded data.

Why is emotional support asked for indirectly, whereas informational support is requested directly? One explanation for the greater use of indirect strategies for soliciting emotional support is that the support may be perceived as less valuable if one needs to ask for it. A major goal of emotional support is to show that the provider cares for the recipient. Simply noticing that the recipient needs support and providing it may itself evidence of caring. Therefore, relatively spontaneous offers of emotional support may be seen as demonstrating more caring than do responses that were explicitly requested by the recipient. In contrast, the value of information or advice is based on the quality of the information offered and not how it was solicited. The quality of this information is not diminished if it is explicitly asked for.

A related explanation is based on the causal attributions people are likely to make if they fail to receive the support they seek. Our data show that people generally receive the type of support they solicited, with moderate correlations between seeking and receiving informational support (*r*=.37) and between seeking and receiving emotional support (*r*=.31). However, people sometimes received support that did not match their intent or received no responses at all. If someone seeks informational support from the community and does not receive it, a plausible attribution is that no one in the community who read the request knew the answer. However, because responses to emotional support requests may be seen as generic expressions of concern, a plausible attribution for failing to receive emotional support when it was requested is that no one in the community cared enough to offer an expression of concern. Thus, requesters might hedge their emotional requests to maintain the illusion that they are “care worthy” even if they fail to receive support and perhaps also to maintain the illusion that other community members are “caring” even if they fail to provide support [43].

A final reason for why people ask explicit questions to elicit informational support but ask for emotional support more implicitly is that people seeking informational support may have an easier task defining what they are looking for than do those seeking emotional support. Informational support seekers want answers to specific questions about treatments, medications, symptoms, and problems of daily living. For example, in our corpus, they wanted to know if it is dangerous to own a cat when one has breast cancer, how long depression lasts after a diagnosis of breast cancer, who are the good doctors in a location, or whether to get reconstructive surgery after breast removal. These people know what kind of information or advice they are seeking and hence can ask for it directly. However, people seeking emotional support may be uncertain about exactly what responses they want. When seeking emotional support, people use phrases such as “I am frightened about the side effects of the chemo and I’m worrying about how it will affect me” or “My friend has just been diagnosed with breast cancer, she is not even 32 years old” or “Terrified of what I have. I am 8 months past radiation and I have this weird swelling on the side of my ribs.” Although these people may have strong needs for comfort, reassurance, or social comparison, they may not be able to articulate the form of support that would relieve their distress. As a result, they may disclose their inner turmoil or the negative events in their lives but fail to request specific types of support because they do not know what to ask for. Instead, they leave it to other members of the community to diagnose their needs and respond appropriately.

## Part 3: Predicting Commitment to a Support Community From Receiving Social Support

### Overview

Although part 2 of the research used machine learning methods with a large data sample to replicate and extend findings from part 1 using a small sample, part 3 of the research shows the value of automated coding by addressing a research question that cannot be practically answered with a small sample—namely, how exposure to support predicts group members’ subsequent participation in the group. Here we briefly summarize a previously published study in which the automated measurement of emotional and informational support described in part 2 was used to predict participants’ length of participation (survival) in their breast cancer support community [25].

Length of participation, an index of commitment to a group, is an important topic for study because it affects the outcomes of individual members as well as the success of the group as a whole. People who stay in an online support community longer are more likely to receive whatever benefits it provides. Moreover, members who remain in the group are resources to fellow members. They share information, provide help, and form social ties with others, and shift over time from being seekers of support to being providers [13,44]. The literature on commitment to communities suggests that both informational and emotional support should increase commitment because participants are likely to consider them important benefits of participation and evidence indicates that received and anticipated benefits increase the likelihood that members will remain in the group [45].

### Methods

#### Overview

We applied survival analysis to test the hypothesis that people who were exposed to more support remained in support communities longer, controlling for the nonsupportive communication they received. Survival analysis is a statistical technique for investigating influences on time-related outcomes, such as whether and when an event occurs. In this study, the event of interest was the time until a member left the community (or conversely, the length of time the person continued participating). The analysis predicted length of participation in support communities from exposure to emotional and information support and other control variables. Because in most online groups the probability of leaving is much higher early in members’ tenure in the group than later on, we used parametric regression survival analysis with time varying independent variables, assuming a Weibull distribution of survival times. The analysis was implemented using the Stata version 13 (StataCorp LP, College Station, TX, USA) streg maximum likelihood estimation for parametric regression survival-time model with multiple records per participant.

#### Sample

The data we collected did not contain information about which messages people read, but only those they posted. To estimate the amount of support people were exposed to, we assumed that people read all the messages in the threads to which they posted in the week they posted. Thus, the survival analysis was limited to the 30,301 people who posted at least 1 message because without overt behavior it was impossible to estimate the amount of support that they viewed. With this volume of data—approximately 30,000 members exposed to approximately half a million messages—it would be impractical to manually code all the posts. Therefore, our measures of exposure were based on the machine-coded measures of emotional and information support described in part 2.

#### Measuring Dropout

We considered the timestamp of the first post by each member as the starting date for participating in the breast cancer discussion forums. Moreover, we assumed that a participant left the community if they failed to post again within 12 weeks of their last post. According to these criteria, a user could drop out from the group and rejoin it multiple times. The results reported subsequently were the same if we assumed that people left the group only once. Because people whose last post was within 12 weeks of the end of data collection could still be participating, we treated them as right censored.

#### Predictor Variables

*Post count exposure* was the total number of posts to which a user was exposed, assuming the user read all the messages posted in threads during the week the user posted. It is the number of posts in the same week and thread in which the user had posted a message.

*Exposure to emotional and informational support* for a user were the mean levels of machine-coded emotional and informational support, respectively, averaged over all messages posted in the same week and thread in which the user had posted a message.

*Has a profile* was a binary measure that indicated whether a user created a profile page (1) or not (0). A total of 31.19% (9,452/30,301) had done so.

*Percent thread starters* was the percentage of an individual’s posts in a week that were thread starters (ie, the number of thread starters a user posted in a week divided by the total number of posts for that user in that week). We included this variable because people who start conversations may be different from those who participate in conversations started by others.

### Results

**Figure 5:**
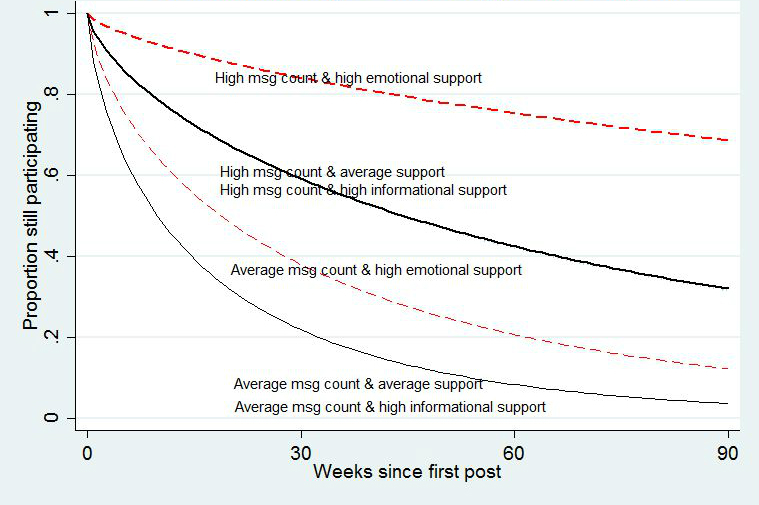
Survival curves for members exposed to different numbers of posts and type of social support. Note: although receiving more informational support was reliably associated with lower longevity on the site, the effect was small and the lines representing high informational support cannot be visually distinguished from the lines representing average informational support.

Table 7 and Figure 5 show the results of the survival analysis. Effects are reported in terms of the hazard ratio, which is the effect of unit increase in an explanatory variable on the probability of participants’ leaving the community in any particular week. Because all explanatory variables except “has a profile” were standardized, the hazard ratio was the predicted change in the probability of dropout for a unit increase in the predictor (ie, has a profile changing from zero to 1 or the continuous variable increasing by a standard deviation when all the other variables are at their mean levels). A hazard ratio greater than 1 indicates an increased probability of leaving, whereas a ratio less than 1 indicates an increased probability of staying.

**Table 7:** Results of the survival analysis.

Predictor variables	Hazard ratio	SE	*P*
Has a profile	0.511	0.010	<.001
% Thread starters	0.853	0.010	<.001
Post count exposure	0.343	0.012	<.001
Emotional support exposure	0.665	0.008	<.001
Informational support exposure	1.048	0.012	<.001
Post count exposure × emotional support exposure	0.493	0.011	<.001
Post count exposure × informational support exposure	0.953	0.020	.02

In interpreting the hazard ratio, the comparison consisted of members with no profiles who were exposed to an average number of messages containing average levels of emotional and informational support. The hazard ratio value of .51 for has a profile meant that members with a profile were 49% more likely to continue to participate than those without a profile (100%–[100%*0.51]). Those who started more threads in a week were 15% more likely to continue participating than those who merely responded to others’ posts.

The hazard ratio for post count exposure indicated that survival rates are 66% higher for those who saw a standard deviation more messages than average, when all other variables were at their average levels. Those who were exposed to an average number of messages containing an average of a standard deviation more emotional support were 33% more likely to remain in the group. In contrast, those who were exposed to messages containing an average number of messages with an average of a standard deviation more informational support were 5% more likely to leave the group. The interaction between number of messages and type of support showed that the differential effects of emotional versus informational support were greater when people were exposed to more messages. Those who were exposed to a standard deviation more messages with a standard deviation more emotional support were 50% more likely to remain in the group, whereas those who were exposed to a standard deviation more messages with a standard deviation more informational support were 5% more likely to leave. The effects of being exposed to more messages and to messages with more emotional support were substantively powerful. For example, based on the survival analysis in Table 7, after 90 days only 3% of those exposed to an average number of messages with an average amount of emotional support would be expected to be active in the groups. In contrast, over 10 times as many (35%) would be expected to be active if they were exposed to a standard deviation more messages at average support levels and over 20 times more (67%) would be expected to be active if they were exposed to a standard deviation more messages containing a standard deviation more than average emotional support.

### Discussion

There are several plausible explanations for why emotional support was associated with remaining in the group whereas informational support was associated with leaving. It may be that many informational needs are short-term. As a result, people who have informational needs and receive information from others have these immediate needs met and have little reason to return, just as one might not continue perusing a dictionary after looking up a definition. On the other hand, the need for emotional support may be longer term and require multiple interactions to be fulfilled. Another possibility is that people who seek emotional support are themselves more social and predisposed to appreciate the encouragement and social comparison the community offers, whereas seekers of information are not. Finally, factual information exchanged in unmoderated health support communities may lack the accuracy, credibility, and usefulness of information from vetted sources, such as physicians or websites run by the American Cancer Society. For this reason, people exposed to informational support may leave because of their negative evaluation of the information they receive. In contrast, emotional support obtained in a community may be perceived as more valuable because the social comparisons, empathy, and encouragement from similar others on the site cannot be duplicated outside the group.

## Summary and Conclusion

The exchange of social support in online support communities is a dynamic communication process in which people actively seek out and then receive support. This study shows that different conversational moves are associated with receiving emotional and informational support in a cancer support community. When people self-disclose about their experiences, especially negative ones, and when they report on their negative thoughts and feelings, others provide them with emotional support. In contrast, when people ask questions and talk about negative but not positive events in their lives, others provide them with informational support. In addition, the support people receive is associated with their continued participation in a support community. They are more likely to continue participating after being exposed to more messages and to messages with higher emotional support, but they are more likely to leave after being exposed to messages with higher informational support.

In addition to these substantive results, our research demonstrated the value of automated coding of online conversations. Most studies of communication in support communities are based on hand coding relatively small samples of conversations. Using machine learning techniques in part 2, we were able to largely replicate findings based on human coding and also to discover relationships that underpowered human-coding studies did not find. In addition, part 3 used automated coding to conduct analyses that would be infeasible with human coding; namely, examining how exposure to more than half a million messages over a 10-year period is associated with over 30,000 participants’ commitment to online support communities.

We also provided an overview of how to use modern machine learning and language analysis techniques to better understand interactions in online health support communities and other online communities. Although previous researchers have shown that it is feasible to automate some text analysis of conversations in online support communities and to produce machine coding results that partially mirror human-coding results, most failed to provide enough details about their methods to allow a skeptical researcher to challenge assumptions they made or to replicate their work. To rectify this shortcoming in the literature, we provided substantial information about machine learning models designed to automatically identify self-disclosure, question asking, and emotional and informational support in breast cancer discussion forums. The performance of our models for predicting social support concepts shows that it is feasible to use computer programs to automatically analyze conversations in online support communities. In particular, we believe that the feature set we used can be fruitfully applied to build predictive models for social support using other health support datasets. LIWC and the linguistic features can be directly applied because they are generic in the sense that they are not tailored to any specific domain. The main work researchers would need to do when applying these methods to another health domain is to recreate the LDA topical dictionaries customized to the data of interest. Many tools now exist for conducting an LDA analysis of a corpus [46,47], and this step can be applied to a new corpus with little effort.

## Limitations and Future Work

Some limitations of the current research should be noted. Regarding the automated coding, accuracy for some constructs, especially the self-disclosure measures, was lower than desirable, thereby attenuating effect sizes. Although it is possible to adjust effect size estimates for attenuation [48], future efforts should be made to improve the measurements, perhaps by adding additional language features or features reflecting the posters’ past behavior. In addition, the ground truth labels for developing the machine learning models were based on annotations provided by MTurk workers who were not the people requesting or receiving support. A natural next step is to validate the labels provided by the MTurk workers with labels provided by the people actually providing or receiving support.

Furthermore, although our current analysis was based on a large corpus of data from 66 forums, we only examined a single disease (breast cancer) in a single online health support site. Other online health support communities might produce different patterns. For example, the positive effect of self-disclosure on emotional support provision in a prostate cancer support community might be different because men are less likely to talk about their thoughts and feelings and, when they do self-disclose, their behavior might be more notable. Further research studying other online support communities can help us better understand and confirm our findings.

Finally, although our findings suggest that people use different language strategies to get informational and emotional social support and that exposure to informational and emotional social support has different effects on commitment, our analyses are correlational. We know that solicitation attempts precede the provision of support and that exposure to support precedes the decision to leave or stay in the group, but we have not demonstrated either that the messages we labeled as support elicitations are actually requesting support, that the messages we labeled as providing support actually do so, or that language in thread-starting messages or replies actually causes others to act differently. Laboratory or field experiments that manipulate support language are needed to test whether the correlational relationships we observed represent causal relationships.

Although the current research used automated coding of support language to better understand the conversational dynamics in health support communities, these same techniques could be used to improve the way these communities function. Using filtering techniques common in online social networking sites, such as Facebook, it would be possible to increase the likelihood that support-seeking messages are seen by those people who are able to provide it. For example, using the automatic classification procedures we have described, it would be possible to automatically identify messages that are seeking a certain type of support but that do not receive it during the 24-hour window when over 80% of messages receive replies. These messages could then be sent by email to others in the community who have been recently active and have responded to similar messages in the past.

## Abbreviations

CFI: comparative fit index
HIT: Human Intelligence Tasks
HR: hazard ratio
LDA: latent Dirichlet allocation
LIWC: Linguistic Inquiry and Word Count
POS: parts of speech
RMSEA: root mean squared error of approximation
